# Effects of increasing soybean meal in corn-based diets on the growth performance of late finishing pigs

**DOI:** 10.1093/tas/txac165

**Published:** 2022-12-13

**Authors:** Julia P Holen, Robert D Goodband, Mike D Tokach, Jason C Woodworth, Joel M DeRouchey, Jordan T Gebhardt

**Affiliations:** Department of Animal Sciences and Industry, College of Agriculture, Kansas State University, Manhattan, KS 66506, USA; Department of Animal Sciences and Industry, College of Agriculture, Kansas State University, Manhattan, KS 66506, USA; Department of Animal Sciences and Industry, College of Agriculture, Kansas State University, Manhattan, KS 66506, USA; Department of Animal Sciences and Industry, College of Agriculture, Kansas State University, Manhattan, KS 66506, USA; Department of Animal Sciences and Industry, College of Agriculture, Kansas State University, Manhattan, KS 66506, USA; Department of Diagnostic Medicine/Pathobiology, College of Veterinary Medicine, Kansas State University, Manhattan, KS 66506, USA

**Keywords:** amino acids, crude protein, finishing pigs, growth performance, soybean meal

## Abstract

Three experiments were conducted to determine the effects of increasing soybean meal (SBM) levels by replacing feed-grade amino acids (AA) in corn, corn dried distillers grains with solubles (DDGS), and corn-wheat midds-based diets on growth performance of late finishing pigs (*n* = 4,406) raised in commercial facilities. Across all experiments, pens of pigs were blocked by initial bodyweight (BW) and randomly assigned to 1 of 5 dietary treatments. All diets were formulated to contain 0.70% standardized ileal digestible (SID) Lys and varying amounts of feed-grade AA. All diets were formulated to meet or exceed minimum essential AA requirement estimates as a ratio to Lys. In Exp. 1, 1,793 pigs (initially 104.9 ± 4.9 kg) were fed corn-based diets and pens of pigs were assigned treatments with increasing SBM from 5% to 20%. Overall, average daily gain (ADG) and feed efficiency (G:F) improved (linear and cubic, *P* ≤ 0.02) as dietary SBM increased, with the greatest improvement observed as SBM increased from 5% to 8.75% and little improvement thereafter. In Exp. 2, 1,827 pigs (initially 97.9 ± 4.3 kg) were fed diets containing 25% DDGS with SBM levels increasing from 0% to 16%. Overall, feed efficiency marginally improved (linear, *P* ≤ 0.10) as SBM increased, with the greatest performance observed when diets contained 8% SBM and similar performance thereafter with 12 or 16% dietary SBM. In Exp. 3, 786 pigs (initially 96.7 ± 3.2 kg) were fed diets that contained 30% wheat midds and dietary SBM from 0% to 16%. Final BW of pigs increased (linear, *P* < 0.05) and overall ADG and G:F improved (linear and cubic, *P* < 0.05) as SBM increased. The combined results of the three experiments suggest that inclusion of at least 4% to 8% dietary SBM at the expense of feed-grade amino acids in corn-based diets with or without grain coproducts can improve growth performance of late-finishing (greater than 100 kg) pigs.

## Teaser

Partial or complete replacement of feed-grade amino acid (AA) with dietary soybean meal in corn-based diets can improve growth performance of late-finishing pigs.

## INTRODUCTION

Soybean meal (SBM) is a key dietary component and commonly used protein source for swine due to its high digestibility, consistent processing methods, and excellent amino acid (AA) profile. However, diets for late-finishing pigs are often formulated to contain increasing amounts of feed-grade AA and grain coproducts such as corn dried distillers grains with solubles (DDGS) and wheat midds to maintain animal growth performance while reducing feed costs and minimizing nitrogen excretion. Due to widespread availability and competitive prices of feed-grade AA, swine diets can be formulated to meet individual AA requirements, resulting in partial or complete replacement of intact protein sources, such as SBM, that also provide nonessential AA. As a result, pigs in the late-finishing phase of growth could consume diets with little to no SBM.

Soto et al. (2019) observed that reductions in CP below 12% may compromise growth performance and carcass characteristics of finishing pigs greater than 100 kg. This suggests some minimal CP requirement, possibly for nonessential AA synthesis. In addition, soybean meal contains biologically active compounds such as isoflavones, saponins, peptides, and omega-3 fatty acids that may be beneficial for immune response and growth performance of pigs exposed to health challenges (Omoni and Aluko, 2005; Smith and Dilger, 2018). Previously, diets with high SBM and reduced feed-grade AA have partially mitigated the negative impact of disease on growth performance of nursery and growing-finishing pigs (Johnston et al., 2010; Rochell et al., 2015).

Although the mechanism for the positive influence of dietary SBM on performance of health-challenged pigs is unclear, the impact of SBM bioactive components must be considered when optimizing growth performance for late-finishing pigs. Additionally, the influence of partial or complete replacement of SBM through inclusion of feed-grade AA and grain coproducts such as corn DDGS and wheat midds must be further evaluated for late-finishing pigs. Therefore, the objective of this experiment was to determine the level of SBM necessary in corn, corn-DDGS, and wheat midds-based diets for optimal growth performance of late-finishing pigs from approximately 100 kg to market.

## MATERIALS AND METHODS

The Kansas State University Institutional Animal Care and Use Committee approved the protocol used in these experiments. A total of three experiments were conducted at commercial research facilities in southwestern Minnesota (New Horizon Farms, Pipestone, MN and New Fashion Pork, Jackson, MN) between November 2019 and December 2020.

### Ingredient chemical analysis

Prior to initiation of Exp. 1 and 3, samples of corn, SBM, and wheat middlings were collected from each feed mill location and submitted to the University of Missouri Agricultural Experimental Station Chemical Laboratories (Columbia, MO) for proximate analyses and complete AA profiles. Standard procedures (AOAC International, 2006) were followed for analysis of amino acid content (method 982.30), moisture (method 934.01), CP (990.03), ether extract (method 920.39), crude fiber (method 978.10), and ash (method 942.05). The analyzed AA content and corresponding AA standard ileal digestibility (SID) coefficients reported by NRC (2012) and proximate analysis values were utilized for diet formulation (Table 1). In Exp. 2, nutrient loading values and SID digestibility coefficients were derived from NRC (2012).

**Table 1.** Average proximate and total amino acid analysis of ingredients (as-fed basis)^*a*,*b*^

Item, %	Corn			Soybean meal			DDGS	Wheat Midds
	Exp. 1		Exp. 3	Exp. 1		Exp. 3	Exp. 2	Exp 3.
	Group 1	Group 2	–	Group 1	Group 2	–	–	–
Dry matter	85.55	86.75	82.08	87.52	88.94	88.27	88.94	87.54
Crude protein	7.90	6.74	6.00	48.48	47.31	45.27	27.74	16.24
Crude fiber	1.57	1.49	1.39	2.85	2.91	4.15	8.12	9.04
Ether extract	1.09	2.39	2.30	0.51	0.49	0.90	6.09	3.40
AA								
Alanine	0.44	0.44	0.44	2.00	2.04	1.93	1.75	0.86
Arginine	0.28	0.28	0.35	3.38	3.40	3.20	1.21	1.24
Aspartic acid	0.43	0.42	0.46	5.34	5.34	4.99	1.74	1.25
Cysteine	0.15	0.14	0.15	0.70	0.70	0.63	0.55	0.37
Glutamic acid	1.10	1.08	1.05	8.70	8.51	7.99	3.39	4.07
Glycine	0.25	0.25	0.28	1.93	1.99	1.92	1.02	0.95
Histidine	0.19	0.18	0.19	1.25	1.27	1.18	0.78	0.48
Isoleucine	0.23	0.24	0.23	2.29	2.35	2.24	1.16	0.59
Leucine	0.71	0.71	0.66	3.66	3.67	3.50	3.17	1.10
Lysine	0.22	0.22	0.27	3.04	3.07	2.93	1.01	0.78
Methionine	0.13	0.13	0.14	0.66	0.67	0.60	0.48	0.25
Phenylalanine	0.31	0.30	0.29	2.49	2.47	2.32	1.46	0.70
Proline	0.51	0.53	0.54	2.35	2.35	2.20	2.08	1.04
Serine	0.29	0.27	0.28	2.03	1.93	1.93	1.17	0.63
Threonine	0.23	0.22	0.22	1.82	1.80	1.69	1.08	0.56
Tryptophan	0.05	0.05	0.05	0.67	0.66	0.64	0.20	0.20
Tyrosine	0.15	0.18	0.19	1.76	1.74	1.61	1.11	0.46
Valine	0.30	0.30	0.31	2.32	2.39	2.27	1.43	0.85

^*a*^A representative sample of each ingredient within experiment was collected and submitted for proximate and amino acid profile analyses to the University of Missouri Experiment Station Chemical Laboratories (Columbia, MO) prior to diet formulation.

^*b*^ NRC (2012) values for proximate and total AA content were used for corn, SBM, and DDGS in Exp. 2 diet formulation as proximate and total AA analyses were not received prior to initiation of the experiment. The DDGS analysis is after the completion of the experiment.

For each experimental diet within each trial, representative diet samples were collected and stored at −20 °C until analysis. Complete diet samples were also analyzed for moisture, CP, ether extract, crude fiber, and ash content as per the standard procedures (Agricultural Experimental Station Chemical Laboratories, University of Missouri, Columbia, MO; Tables 2–4).

**Table 2.** Ingredient and nutrient composition of diets, Exp. 1 (as-fed basis)

Ingredient, %	Soybean meal, %				
	5.00	8.75	12.50	16.25	20.00
Corn	90.68	87.28	83.93	80.51	76.98
Soybean meal (47% CP)	5.00	8.75	12.50	16.25	20.00
Choice white grease	0.85	0.95	1.00	1.00	1.05
Calcium carbonate	0.80	0.80	0.77	0.77	0.77
Monocalcium phosphate (21.5% P)	0.65	0.60	0.55	0.50	0.45
Salt	0.50	0.50	0.50	0.50	0.50
L-Lys-HCl	0.53	0.41	0.28	0.16	0.04
DL-Met	0.16	0.12	0.08	0.05	0.01
L-Thr	0.22	0.17	0.12	0.07	0.02
L-Trp	0.07	0.05	0.03	0.01	–
L-Val	0.18	0.11	0.04	–	–
L-Ile^*a*^	0.15	0.07	0 or 0.01	–	–
His^*a*^	0.03	0 or 0.01	0 or 0.01	–	–
Vitamin trace mineral premix^*b*^	0.15	0.15	0.15	0.15	0.15
Phytase^*c*^	0.03	0.03	0.03	0.03	0.03
Total	100.00	100.00	100.00	100.00	100.00
Calculated nutrient analysis					
Standardized ileal digestible (SID) AA, %					
Lys	0.70	0.70	0.70	0.70	0.70
Ile:Lys	60	60	60	69	79
Leu:Lys	103	117	132	146	160
Met:Lys	40	38	35	33	30
Met and Cys:Lys	60	60	60	60	60
Thr:Lys	65	65	65	65	65
Trp:Lys	20.0	20.0	20.0	20.0	21.6
Val:Lys	72	72	72	75	85
His:Lys	32	34	39	44	49
SID Lys:NE, g/Mcal	2.62	2.62	2.62	2.62	2.62
NE, kcal/kg	2,665	2,665	2,665	2,665	2,665
CP, %	10.1	11.3	12.6	13.9	15.2
Ca, %	0.48	0.48	0.48	0.48	0.48
STTD P, %^*d*^	0.33	0.33	0.33	0.33	0.33
Analyzed composition, %^*e*^					
DM	85.8	87.5	87.4	87.2	87.5
CP	11.0	11.8	12.5	12.3	14.1
Crude fat	2.4	2.7	2.5	2.8	3.2
Crude fiber	1.8	2.5	2.0	2.1	2.3

^*a*^Range of values reflect changes in chemical analysis of ingredients in groups one and two, respectively.

^*b*^Provided the following nutrients per kg of premix: 3,527,360 IU vitamin A; 881, 840 IU vitamin D; 17,637 IU vitamin E, 1,764 mg vitamin K, 15.4 mg vitamin B_12_, 33,069 mg niacin; 11,023 mg pantothenic acid; 3,307 mg riboflavin, 74 g Zn from Zn sulfate; 74 g Fe from Fe sulfate; 22 g Mn from Mn oxide; 11 g Cu from Cu sulfate; 0.22 g I from calcium iodate; 0.20 g Se from sodium selenite.

^*c*^Optiphos 2000 PF (Huvepharma Inc. Peachtree City, GA).

^*d*^STTD P = standardized total tract digestible phosphorus.

^*e*^A composite sample of each treatment was collected and submitted to the University of Missouri Agricultural Experiment Station Chemical Laboratories (Colombia, MO) for proximate analysis.

**Table 3.** Ingredient and nutrient composition of diets, Exp. 2 (as-fed basis)^*a*^

Ingredient, %	Soybean meal, %				
	0	4	8	12	16
Corn	71.39	67.67	63.92	60.11	56.28
Corn DDGS (7.5% oil)	25.00	25.00	25.00	25.00	25.00
Soybean meal (47% CP)	–	4.00	8.00	12.00	16.00
Beef tallow	1.00	1.00	1.00	1.00	1.00
Calcium carbonate	0.95	0.95	0.95	0.97	0.97
Monocalcium phosphate (21.5% P)	0.20	0.15	0.10	0.05	–
Salt	0.50	0.50	0.50	0.50	0.50
L-Lys-HCl	0.57	0.45	0.32	0.19	0.07
L-Thr	0.12	0.06	0.01	–	–
L-Trp	0.06	0.04	0.02	–	–
L-Val	0.01	–	–	–	–
L-Ile	0.02	–	–	–	–
Vitamin trace mineral premix^*b*^	0.15	0.15	0.15	0.15	0.15
Phytase^*c*^	0.03	0.03	0.03	0.03	0.03
Total	100.00	100.00	100.00	100.00	100.00
Calculated nutrient analysis					
Standardized ileal digestible (SID) AA, %					
Lys	0.70	0.70	0.70	0.70	0.70
Ile:Lys	55	62	71	81	91
Leu:Lys	182	196	209	223	237
Met:Lys	32	34	37	40	42
Met and Cys:Lys	61	66	71	76	82
Thr:Lys	65	65	65	72	79
Trp:Lys	19.5	19.5	19.5	19.9	23.1
Val:Lys	70	78	88	97	107
His:Lys	41	46	52	57	63
SID Lys:NE, g/Mcal	2.68	2.68	2.68	2.68	2.68
CP, %	13.6	15.0	16.4	17.9	19.4
NE, kcal/kg	2,611	2,611	2,611	2,611	2,611
Ca, %	0.47	0.48	0.48	0.49	0.50
STTD P, %^*d*^	0.33	0.33	0.33	0.33	0.33
Analyzed composition, %^*e*^					
DM	87.0	87.5	87.4	87.2	87.5
CP	13.7	13.2	14.9	17.4	19.1
Crude fat	6.5	4.3	4.2	4.5	4.6
Crude fiber	4.1	2.7	2.9	3.4	3.2

^*a*^Experimental diets were fed from 97.9 kg to market.

^*b*^Provided the following nutrients per kg of premix: 3,527,360 IU vitamin A; 881, 840 IU vitamin D; 17,637 IU vitamin E, 1,764 mg vitamin K, 15.4 mg vitamin B_12_, 33,069 mg niacin; 11,023 mg pantothenic acid; 3,307 mg riboflavin, 74 g Zn from Zn sulfate; 74 g Fe from Fe sulfate; 22 g Mn from Mn oxide; 11 g Cu from Cu sulfate; 0.22 g I from calcium iodate; 0.20 g Se from sodium selenite.

^*c*^Optiphos 2000 PF (Huvepharma Inc. Peachtree City, GA).

^*d*^STTD P = standardized total tract digestible phosphorus.

^*e*^A composite sample of each treatment diet was collected and submitted to the University of Missouri Agricultural Experiment Station Chemical Laboratories (Colombia, MO) for proximate analysis.

**Table 4.** Ingredient and nutrient composition of diets, Exp. 3 (as-fed basis)^*a*^

Ingredient, %	Soybean meal, %				
	0	4	8	12	16
Corn	65.58	61.95	58.35	54.64	50.90
Wheat middlings	30.00	30.00	30.00	30.00	30.00
Soybean meal (47% CP)	–	4.00	8.00	12.00	16.00
Choice white grease	1.00	1.05	1.11	1.13	1.11
Calcium carbonate	1.28	1.28	1.25	1.25	1.25
Monocalcium phosphate (21.5% P)	0.30	0.25	0.20	0.15	0.10
Salt	0.50	0.50	0.50	0.50	0.50
L-Lys-HCl	0.49	0.37	0.25	0.12	–
DL-Met	0.12	0.09	0.05	0.02	0.01
L-Trp	0.06	0.04	0.01	–	–
L-Val	0.13	0.06	–	–	–
L-Ile	0.16	0.09	0.03	–	–
Thr^*b*^	0.28	0.22	0.15	0.09	0.03
Vitamin trace mineral premix^*c*^	0.10	0.10	0.10	0.10	0.10
Total	100.00	100.00	100.00	100.00	100.00
Calculated nutrient analysis					
Standardized ileal digestible (SID) AA, %					
Lys	0.70	0.70	0.70	0.70	0.70
Ile:Lys	60	61	62	69	79
Leu:Lys	91	106	120	135	150
Met:Lys	37	34	32	30	30
Met and Cys:Lys	60	60	60	60	63
Thr:Lys	65	65	65	65	65
Trp:Lys	19.5	19.5	19.5	20.0	23.1
Val:Lys	70	71	73	83	93
His:Lys	32	37	42	48	53
SID Lys:NE, g/Mcal	2.81	2.81	2.81	2.81	2.81
CP, %	9.8	11.1	12.4	13.8	15.2
NE, kcal/kg	2,502	2,502	2,502	2,502	2,502
Ca, %	0.60	0.60	0.60	0.60	0.60
STTD P, %^*d*^	0.28	0.28	0.28	0.28	0.28
Analyzed composition, %^*e*^					
DM	86.7	86.9	86.9	86.5	86.5
CP	11.1	12.4	14.1	14.7	16.4
Crude fat	3.82	4.03	3.90	3.57	3.63
Crude fiber	3.64	3.64	4.01	4.02	3.57

^*a*^Experimental diets were fed from 96.7 kg to market.

^*b*^L-Threonine 80% with BioMass (CJ America Bio, Downers Grove, IL).

^*c*^Provided the following nutrients per kg of premix: 4,729,048 IU vitamin A; 207,077 IU vitamin D_3_; 21,650 IU vitamin E; 1,792 mg riboflavin; 9,911 mg niacin; 6,577 mg pantothenic acid; 930 mg vitamin K; 60 mg Zn; 37.5 mg Fe; 12 mg Mn; 9 mg Cu; 0.2 mg I; 0.2 mg Se.

^*d*^STTD P = standardized total tract digestible phosphorus.

^*e*^A composite sample of each treatment was collected and submitted to the University of Missouri Agricultural Experiment Station Chemical Laboratories (Colombia, MO) for proximate analysis.

### Animals and diets

Experiments 1 and 2 were conducted at a commercial research facility in southwestern Minnesota (New Horizon Farms, Pipestone, MN). The facility was a naturally ventilated and double-curtained-sided barn. Each pen (3.0 × 5.5 m) was equipped with a four-hole stainless steel dry feeder (Thorp Equipment Inc., Thorp, WI) and one cup waterer to allow ad libitum access to feed and water. Experiment 3 was completed in a tunnel-ventilated barn with completely slatted flooring over deep pits for manure storage (New Fashion Pork, Jackson, MN). Each pen (2.4 × 5.8 m) was equipped with a three-hole stainless steel dry feeder (Thorp Equipment Inc., Thorp, WI) and a pan waterer to allow ad libitum access to feed and water. For all experiments, daily feed additions for each pen were recorded through a robotic feeding system (FeedPro, Feedlogic Corp., Wilmar, MN).

In Exp. 1, there were two groups of approximately 900 pigs for a total of 1,793 pigs (L337 × 1050, PIC, Hendersonville, TN; initially 104.9 ± 4.9 kg) used. The pigs in each group were housed in 2 identical barns within the 4-barn facility. The two groups were started on test approximately 6 months apart, therefore we collected and analyzed major ingredients before each group and adjusted diet formulation slightly. There were 22 to 27 pigs placed per pen with equal numbers of pigs within a block and 12 to 14 pens per treatment. Two pens had to be removed from the data set due to computerized feeding system breakdown. Pens of pigs were blocked by initial BW and randomly assigned to 1 of 5 dietary treatments in a randomized complete block design. Soybean meal levels within corn-based diets gradually increased from 5% to 20% in 3.75% increments, replacing feed-grade AA. All dietary treatments were formulated to be isocaloric and contained 0.70% SID Lys (Table 2). Furthermore, additions of feed-grade AA were adjusted to meet or exceed the minimum essential AA requirements in relation to Lys. to ensure similar Ile, Met & Cys, Thr, Trp, Val, and His concentrations among treatments between the two experimental groups. The NE of SBM used in diet formulation was 2,672 kcal/kg (as-fed basis) to represent 100% of the corn NE reported by NRC (2012; Cemin et al., 2020). Pens of pigs were weighed and feed disappearance measured on d 0, 13, and 23 or on d 0, 14, and 35 for groups one and two, respectively, to determine average daily gain (ADG), average daily feed intake (ADFI), and feed efficiency (G:F). On d 13 and 21 of the experimental period for groups one and two, respectively, three pigs within each pen were marketed. The remaining pigs were then marketed at the conclusion of the experiment.

In Exp. 2, there were two groups of approximately 900 pigs for a total of 1,827 pigs (L337 × 1050, PIC; initially 97.9 ± 4.3 kg) used. There were 23 to 27 pigs per pen and 14 pens per treatment. Like in Exp. 1, the pigs in each group were housed in 2 identical barns within the 4-barn facility. The two groups were started on test approximately 1 month apart. Pens of pigs were blocked by initial BW and randomly assigned to 1 of 5 dietary treatments in a randomized complete block design. Experimental diets were corn-based with 25% DDGS and feed-grade AA. Soybean meal levels increased from 0% to 16% in 4% increments and replaced feed-grade AA. All diets were formulated using assumed ingredient AA composition and SID from the NRC (2012). Furthermore, the NE of SBM used in diet formulation was 2,672 kcal/kg (as-fed basis) to represent 100% of the corn NE reported in the NRC (2012). The DDGS were assumed to contain 91% of the NE of corn or 2,432 kcal/kg. Diets were formulated to be isocaloric and contained 0.70% SID Lys (Table 3). Dietary additions of feed-grade AA were adjusted to meet or exceed the minimum essential AA requirements in relation to Lys. Pens of pigs were weighed, and feed disappearance measured on d 0, 15, and 29 or on d 0, 19, 34, and 42 for groups one and two, respectively, to determine ADG, ADFI, and G:F. Additionally, two pigs within each pen were marketed on d 15 and 19 of the experimental period for groups one and two, respectively. The remaining pigs were marketed at the conclusion of the experiment. Due to slower growth performance of pigs within group 2, the experimental period was extended from 34 d to 42 d to achieve similar final BW between the two studies.

In Exp. 3, a total of 786 pigs (PIC TR4 × (Fast LW × PIC L02); initial BW = 96.7 ± 3.2 kg) were used in a 40-d trial with 15 to 19 pigs per pen and 9 pens per treatment. Pens of pigs were blocked by initial BW and randomly assigned to 1 of 5 dietary treatments in a randomized complete block design. Dietary treatments were corn-based with 30% wheat midds and feed-grade AA. As in Exp. 2, soybean meal levels increased from 0% to 16% in 4% increments and replaced feed-grade AA. The analyzed nutrient compositions of ingredients were utilized in the diet formulation such that all diets were isocaloric and contained 0.70% SID Lys (Table 4). Dietary additions of feed-grade AA were adjusted to meet or exceed the minimum essential AA requirements in relation to Lys. Additionally, the NE of SBM used in diet formulation was 2,672 kcal/kg (as-fed basis) to represent 100% of the corn NE reported in the NRC (2012). Pens of pigs were weighed and feed disappearance measured on d 0, 20, 32, and 40 to determine ADG, ADFI, and G:F.

### Statistical analysis

Data were analyzed using the GLIMMIX procedure in SAS (Version 9.4, SAS Institute, Inc., Cary, NC) with pen as the experimental unit. Where experiments used 2 groups of pigs, treatment by group interactions were tested and found not to be significant, therefore the data were combined. The statistical model considered fixed effects of dietary treatment and random effects of group and block. Means were separated with linear, quadratic, and cubic contrasts. All data are reported as least square means and considered statistically significant at *P* ≤ 0.05 and marginally significant at 0.05 < *P* ≤ 0.10.

## RESULTS

### Experiment 1

Increasing dietary SBM from 5% to 20% in corn-based diets improved overall ADG from 0.74 kg to 0.83 kg and G:F from 265 to 302 g/kg for pigs in the late-finishing phase of growth (linear, *P* ≤ 0.001; Table 5). Interestingly, the observed improvements in overall ADG and feed efficiency were not only linear with increasing SBM, but there was also evidence for a cubic response (*P* < 0.05). The greatest improvements in growth performance were initially observed as SBM levels increased from 5% to 8.75% but then further improved as dietary SBM was increased from 16.25% to 20%. However, final BW of pigs were similar regardless of dietary SBM level (*P* > 0.05).

**Table 5.** Effects increasing levels of soybean meal in corn-based diets on late-finishing pig growth performance, Exp. 1^*a*^

Item	Soybean meal, %						Probability, *P* =		
	5.00	8.75	12.50	16.25	20.00	SEM	Linear	Quadratic	Cubic
BW^*b*^, kg									
Initial	105.4	104.9	104.6	104.8	104.8	1.41	0.592	0.644	0.906
Final	124.8	127.1	126.3	126.3	127.2	1.38	0.129	0.490	0.125
Overall									
ADG, kg	0.74	0.82	0.80	0.80	0.83	0.017	0.001	0.142	0.011
ADFI, kg	2.80	2.85	2.79	2.79	2.75	0.036	0.118	0.331	0.624
G:F, g/kg	265	287	288	287	302	6.7	< 0.001	0.373	0.020

^*a*^A total of 1,793 pigs (L337 × 1050, PIC) were used with 22 to 27 pigs per pen and 12 to 14 replications per treatment.

^*b*^BW = body weight.

### Experiment 2

When provided corn-based diets that included 25% DDGS, increasing dietary SBM from 0% to 16% did not influence final BW of pigs in the late-finish period of growth (Table 6; *P* > 0.05). Although there was no evidence for differences among treatments with increasing SBM levels on overall ADG or ADFI (*P* > 0.05), a tendency for improved overall feed efficiency was observed as dietary SBM levels increased from 0% to 16% (linear, *P* = 0.100).

**Table 6.** Effects of increasing levels of soybean meal in corn and soybean meal-based diets containing 25% DDGS on late-finishing pig growth performance, Exp. 2^*a*^

Item	Soybean meal, %						Probability, *P* =		
	0	4	8	12	16	SEM	Linear	Quadratic	Cubic
BW^*b*^, kg									
Initial	98.1	97.8	97.8	97.8	98.0	1.15	0.661	0.218	0.735
Final	124.1	124.3	125.3	124.4	124.0	0.81	0.960	0.107	0.865
Growth performance									
ADG, kg	0.77	0.76	0.80	0.79	0.78	0.023	0.317	0.231	0.321
ADFI, kg	2.65	2.61	2.66	2.64	2.61	0.080	0.637	0.594	0.188
G:F, g/kg	291	294	300	298	299	4.2	0.100	0.380	0.957

^*a*^A total of 1,827 pigs (L337 × 1050, PIC) were used in 2 groups with 23 to 27 pigs per pen and 14 replications per treatment.

^*b*^BW = body weight.

### Experiment 3

Overall, increasing dietary SBM from 0% to 16% in diets containing 30% wheat midds improved ADG for pigs during the late-finishing phase of growth (linear, *P* = 0.005; Table 7). Additionally, feed efficiency improved as SBM increased from 0% to 16% (linear, *P* < 0.001). These advantages in growth performance resulted in heavier final BW of pigs as dietary SBM levels increased from 0% to 16% (linear, *P* < 0.05). The observed advantages in overall ADG and G:F not only correlated linearly with increasing SBM, but cubic effects were also observed (*P* < 0.05). Initial increases in growth performance were observed as SBM increased from 0% to 4% of the diet and then further improved from 12% to 16% of the diet.

**Table 7.** Effects of increasing soybean meal in corn and soybean meal-based diets containing 30% wheat midds on late-finishing pig growth performance, Exp. 3^*a*^

Item	Soybean meal, %						*P *=		
	0	4	8	12	16	SEM	Linear	Quadratic	Cubic
BW^*b*^, kg									
Initial	96.1	96.7	96.8	97.3	96.7	1.08	0.213	0.244	0.809
Final	129.7	131.3	131.1	130.6	133.4	1.41	0.042	0.620	0.113
Growth performance									
ADG, kg	0.97	1.00	1.01	0.98	1.06	0.018	0.005	0.396	0.032
ADFI, kg	3.39	3.44	3.42	3.39	3.49	0.051	0.358	0.615	0.214
G:F, g/kg	285	291	295	290	305	3.6	< 0.001	0.495	0.035

^*a*^A total of 786 pigs (PIC TR4 × [Fast LW × PIC L02]) were used in a 40-d experiment with 15 to 19 pigs per pen and 9 replications per treatment.

^*b*^BW = body weight.

### Removals and Mortality

Throughout all three experiments, pigs did not exhibit clinical health challenges. Removal rate was 2.3%, 1.4%, and 0.2%, and mortality rate before removal was 0.3%, 0.0%, and 0.2% in experiments 1, 2, and 3, respectively. Common reasons for removal included lameness, and belly ruptures.

## DISCUSSION

Soybean meal is largely considered the standard plant protein (amino acid) source in swine diet formulation to complement cereal grains in corn or wheat-based diets that may otherwise be deficient in AA. In the United States, dehulled, solvent extracted SBM is often utilized in swine diet formulation due to its high CP content, balanced AA profile, and high digestibility of essential AA ranging from 85% to 94% (NRC, 2012).

With increased commercial availability, feed-grade sources of Lys, Met, Thr, Trp, and Val are often incorporated in swine diets to either partially or completely replace intact protein sources such as SBM. This offers reduced diet costs and limits excess nitrogen excretion. Previous studies show that growth performance and carcass composition of growing-finishing pigs provided diets with reduced CP and high levels of feed-grade AA are similar to pigs fed diets with high CP provided by SBM (Hinson et al., 2009; Tous et al., 2014; Molist et al., 2016). However, recent evaluation of diets formulated to meet NRC (2012) AA requirements but with a linear CP reduction from 13% to 9% of the diet suggests that late-finishing pigs (greater than 100 kg) require at least 12% to 13% dietary CP to prevent decreased gain and feed efficiency (Soto et al., 2019). Additionally, it is unclear if beneficial growth responses for late-finishing pigs in response to greater dietary CP are a result of inclusion of intact protein sources that provide greater nonessential AA concentrations. In Exp. 1 and 3, the diets with little to no SBM were below 12% CP. Therefore, the responses observed herein might be similar as Soto et al. (2019), where it appears a minimum level of approximately 12% CP is needed.

A second possible explanation for our results is that soybean meal contains biologically active compounds such as isoflavones, saponins, phytosterols, and omega-3 fatty acids that possess antiviral, anti-inflammatory, and antioxidant properties that can positively influence immune response and growth performance of pigs (Omoni and Aluko, 2005; Smith and Dilger, 2018). The positive influence of SBM beyond its nutritive role was first recognized by Boyd et al. (2010) in a study monitoring disease-challenged growing pigs. Improved final market BW, ADG, and feed efficiency were observed when finishing pigs were provided corn-SBM-based diets compared to low SBM diets supplemented with feed-grade AA (Boyd et al., 2010). The advantages of dietary SBM have also been observed among disease-challenged nursery pigs. Studies on porcine respiratory and reproductive syndrome (PRRS)-positive nursery pigs have also observed improved feed efficiency when dietary SBM increased from 12.5% to 22.5% (Rocha et al., 2013) or from 17.5% to 29% (Rochell et al., 2015). However, increasing dietary SBM for healthy nursery pigs did not influence growth performance (Rochell et al., 2015).

As stated in a review by Smith and Dilger (2018), bioactive compounds of SBM such as soy isoflavones that provide anti-inflammatory, antioxidative, and antiviral properties may improve the immunological status and growth of pigs exposed to disease challenges but may not benefit healthy pigs. A recent study with nursery pigs observed greater ADFI and final BW in pigs provided corn-SBM diets compared with soy protein concentrate-based diets plus pure soy isoflavones (Li et al., 2020). Additionally, four experiments conducted by Cemin et al. (2020) with healthy nursery pigs observed consistent linear improvements in feed efficiency as SBM increased from 27.5% to 37.5%. Among wean-to-finish pigs, however, there was no evidence for differences in growth performance when pigs were provided diets with either SBM or soy protein concentrate (Kuhn et al., 2004). Furthermore, dietary inclusion of pure isoflavones in soy protein concentrate-based diets was observed to negatively influence growth performance of late-finishing pigs when compared to SBM-based diets (Payne et al., 2001). Although there are other bioactive compounds in SBM, it is not clear why elevated levels of soy isoflavones in diets positively influence growth performance in some pig populations but not others.

In Exp. 1 of the present study that evaluated corn-SBM based diets with increasing SBM from 5% to 20%, linear improvements in overall ADG and feed efficiency were observed. As SBM increased, CP also increased from 11% to 14%, which may have contributed to the observed advantage in growth performance. The results of our study align with those of Anderson (2021), where increased supplementation of feed-grade AA and reduced SBM in corn-based diets provided to finishing pigs (initial BW = 83.1 kg) linearly reduced overall ADG and feed efficiency of the pigs.

Increased dietary SBM from 0% to 16% in diets that contained 25% DDGS in Exp. 2 marginally improved feed efficiency of late-finishing pigs in the present study but did not influence final BW or ADG. Cemin et al. (2021) observed a similar response where increasing feed-grade Lys at the expense of dietary SBM in diets containing 10% DDGS resulted in a quadratic decrease in feed efficiency of late-finishing pigs. In Exp. 2, analyzed CP of all dietary treatments exceeded the 13% CP requirement estimate established by Soto et al. (2019). However, diets within the Anderson (2021) study contained 20% DDGS and linear reductions in CP from approximately 18.5% to 11.8% across the late-finishing period. Therefore, it is possible that late-finishing swine diets with lower DDGS levels and CP may benefit from increasing SBM.

Wheat middlings have a much lower CP content (15.8%) than corn DDGS (27.4%; NRC, 2012). As a result, inclusion of 30% wheat middlings in Exp. 3 resulted in diets containing 11.1% to 16.4% CP as SBM levels increased from 0% to 16%. Similar to Exp. 1, advantages in growth performance were observed for pigs provided diets with increasing SBM. However, not only were the observed improvements in overall ADG and feed efficiency linear with increasing SBM, but there was also evidence for cubic responses in both Exp. 1 and 3. We have no explanation for a cubic response to increasing SBM levels in the diet. However, it may be due to the greater SID Trp:Lys ratios in corn-based diets that contained 20% SBM, or corn-wheat midds-based diets that contained 16% SBM. While increasing SID Trp:Lys ratio above 20% has not consistently increased growth performance of growing-finishing pigs (Soto et al., 2017; Gonçalves et al., 2018; Williams et al., 2020), it may be a factor for the cubic responses to increased SBM in Exp. 1 and 3.

## CONCLUSIONS

The results of these experiments suggest that increasing dietary SBM to partially or fully replace feed-grade AA can benefit growth performance of late-finishing pigs (greater than 100 kg). Although the specific biological mechanism responsible for the advantages in performance is unknown, it might be related to either the CP content of the diet or bio-active compounds in SBM. These results suggest that corn-SBM based diets should contain at least 8% SBM to optimize ADG and feed efficiency. Among diets that contained 25% DDGS, dietary SBM level did not appear to influence growth but did tend to improve feed efficiency of late-finishing pigs. For late-finishing pigs fed corn-SBM based diets with 30% wheat midds, inclusion of at least 4% SBM can improve ADG and feed efficiency. Further research to understand the cubic response to the highest SBM inclusion rate in corn-SBM or corn-SBM-wheat midds based diets in these experiments is warranted.

## Abbreviations

AA: amino acid
ADG: average daily gain
ADFI: average daily feed intake
BW: body weight
CP: crude protein
DDGS: dried distillers grains with solubles
DM: dry matter
G:F: feed efficiency
HCW: hot carcass weight
NE: net energy
SBM: soybean meal
SID: standardized ileal digestible
